# The Implication of microRNAs as non-invasive biomarkers in 179 Egyptian breast cancer female patients

**DOI:** 10.32604/or.2022.027277

**Published:** 2023-02-09

**Authors:** NADIA Z. SHAABAN, NASHWA K. IBRAHIM, HELEN N. SAADA, FATMA H. EL-RASHIDY, HEBATALLAH M. SHAABAN, NERMEEN M. ELBAKARY, AHMAD S. KODOUS

**Affiliations:** 1 Biochemistry Department, Faculty of Science, Alexandria University, P.O. Box 21568, Alexandria, Egypt; 2 Radiation Biology Department, National Center for Radiation Research & Technology, Egyptian Atomic-Energy Authority, P.O. Box 8029, Cairo, Egypt; 3 National Cancer Institute, Cairo University, P.O. Box 11796, Cairo, Egypt

**Keywords:** miR-21 and miR-34a, Breast-cancer, BRCA1 and BRCA2, Bcl-2, p53

## Abstract

**Background:**

MicroRNAs (miRs) are small (19–25 nucleotides), non-protein coding RNAs that regulate gene expression, and thus play essential roles in cell cycle progression. The evidence has demonstrated that the expression of several miRs is dysregulated in human cancer.

**Methods:**

The study includes 179 female patients and 58 healthy women Patients were identified as luminal A, B, Her-2/neu, and basal-like, as well as classified into I, II, and III stages. Analysis of the expression fold change of miR-21 and miR-34a with molecular markers, including the oncogene Bcl-2 (B-cell lymphoma 2) and the tumor suppressor genes BRCA1 (breast cancer susceptibility gene 1), BRCA2 (breast cancer susceptibility gene 2), and the tumor suppressor protein p53, was carried out for all patients, pre- and post-chemotherapy, and for all healthy women.

**Results:**

At diagnosis (pre-chemotherapy), miR-21 was up-regulated (*p* < 0.001), while miR-34a was down-regulated (*p* < 0.001). Post-chemotherapy, the expression of miR-21 decreased significantly (*p* < 0.001), while the expression of miR-34a increased significantly (*p* < 0.001).

**Conclusion:**

miR-21 and miR-34a may be helpful to non-invasive biomarkers to evaluate the response of breast cancer to chemotherapy.

## Key Points:

• In breast cancer, the expression of miR-21 and miR-34a is altered.

• miR-21 correlates directly with the oncogene Bcl2,

• miR-34a correlates directly with the tumor suppressor protein p53

• miR-34a correlates directly with breast-cancer susceptibility genes BRCA1, and BRCA2.

• miR-21 and miR-34a could predict the response of breast cancer to chemotherapy

## Background

Breast cancer (BC) is still the most common malignancy in women and has a high incidence of mortality globally [1–3]. As it represents 32% of all Egyptian female malignancies, it is considered the most prevalent type of cancer in Egypt [4,5]. BC is a complex of different cellular origins, etiology, and response to treatment [6]. The existence or absence of molecular markers for epidermal growth factor 2 and estrogen or progesterone receptors divides BC into major BC subtypes. The currently applied treatments for BC roughly depend on the tumor subtype and anatomic stage of cancer. These treatments include chemotherapy, radiation, modulators of hormone receptors, immunotherapy and endocrine therapy [7].

Different treatments have led to better results; nonetheless, some forms of BC exhibit tumor progression due to drug resistance, DNA repair pathways, apoptotic resistance, and miRNAs Marquette et al. [8] reported as possible indicators of response to different BC treatments [9]. The miRNAs are small non-protein coding RNAs of 19–25 nucleotides that play essential roles in the control of the cell cycle by modulating the expression of different cell cycle controllers [10]. Under normal conditions, miRNAs bind to their target mRNAs’ untranslated regions (3′-UTRs) directly and thus regulating their translation and stability.

The evidence has demonstrated that the miRNAs of clinical importance in BC are miR-21 and miR-34a [11]. The miR-21, identified as an oncomir that disrupts apoptosis, was reported to be up-regulated in BC. On the other hand, miR-34a, a pivotal member of the Tumor suppressor p53 (p53) network that induces apoptosis, was shown to be down-regulated in BC [12,13]. It is well documented that the initiation, progression and metastasis of BC are caused by multiple genetic alterations. The majority of studies have focused on the role of oncogenes such as Bcl-2 (B-cell lymphoma 2) and tumor suppressor genes as the breast cancer susceptibility gene 1 (BRCA1), the breast cancer susceptibility gene 2 (BRCA2), and the tumor suppressor protein p53. In this work, we looked at the levels of miR-21 and miR-34a in parallel to Bcl-2, BRCA1, BRCA2, and p53 in female BC patients before and after chemotherapy to evaluate how they responded to the chemotherapy treatment.

## Methods

### Blood

Blood was drawn from 179 BC patients (average age 52 years ranging from 28.5–75), pre and post-chemotherapy. All patients had confirmed primary BC diagnosis histologically. Blood samples were also collected from 58 healthy women (median age 53.5 years ranging from 32–71). The National Cancer Institute (NCI) Hospital’s Ethic Committee authorized the research following the 1964 Declaration of Helsinki, and all patients signed permission.

### Tissue preparation

For morphological examination, paraffin-embedded tissue blocks were stained with haematoxylin-eosin [14]. According to the WHO 2003 categorization of breast tumors [15,16], tumor grade was calculated using the Elston and Ellis grading method [17], and tumor stage was evaluated using TNM [15,16]. The WHO/ISUP of 2004 classified tumors as Luminal A, Luminal B, Basal-Like, and HER2/neu.

### Chemotherapy

The neo-adjuvant chemotherapy protocol included 4–6 cycle chemotherapy concurrent given q3 weeks. FAC: 5-Fluorouracil, Adriamycin, Cyclophosphamide. FEC: 5-Fluorouracil, Epirubicin and Cyclophosphamide. CMF: Cyclophosphamide, Methotrexate, 5-Fluorouracil. TE: Paclitaxel and Epirubicin.

### Immunohistochemistry

The streptoavidin-biotin immunoperoxidase technique was used for immunohistochemical staining (Dakocytomation, Glostrup, Denmark). Monoclonal antibodies against ER, PR, and HER2 receptors (Santa Cruz Biotechnology, CA) and 2^nd^ antibodies were used to assay hormone receptors.

### Extraction of RNA

Blood was drawn, and TRIZOL-RNA isolation protocol was used to extract total RNA from it [18]. RNA sample quality was determined by Nanodrop. Genomic DNA contamination must be eliminated [18,19]. Small RNA molecules were obtained using kits (Qiagen, GmbH, Hilden, Germany) [20], followed by the addition of miRNA, acid-phenol and chloroform, then centrifugation [21]. Even if the filter cartridge includes large RNA, absolute ethanol was added, and the filtrate contains small RNA; after, small and large RNAs were collected [20,21].

### miR-21 and miR-34a expressions

It was estimated by Applied Biosystems 7500 Real-time PCR System (Applied Biosystems; USA), and the housekeeping miRNA U6 RNA, as well as a 96-well miScript miRNA PCR plate with forward and reverse miRNA specific primers, according to the manufacturer’s instructions (QIAGEN GmbH, Hilden, Germany). 95°C for 15 min, then 40 cycles of 95°C for 5 s and 60°C for 34 s. The expression of miRNA was calculated using the cycle threshold (Ct) technique [22,23]. miRNA expression levels were determined using the ΔΔCt technique for relative quantification of gene expression [24]. The equation 2^−ΔΔCt^ was used to calculate the fold change [11,22,23,25].

### Real-time PCR for p53, Bcl-2, BRCA1, and BRCA2 expressions

For each gene, all reactions were performed in triplicate and contained no template or reverse transcription controls. For 40 cycles, 15 s at 95°C and 1 min at 60°C were used as cyclic conditions. The ΔΔCt technique was used to determine the levels of mRNA expression. To standardize their relative abundance, Taqman mRNA Assays for GAPDH (glyceraldehyde-3-phosphate dehydrogenase) were utilized [11].

### Statistical analysis

Data was analyzed using IBM SPSS advanced statistics version 22 (SPSS Inc., Chicago, IL). Data were stated as mean ± standard deviation. Qualitative data were expressed as frequency and percentage. Mann–Whitney test (non-parametric *t*-test) was used to compare between two groups. Comparison between more than two groups was made using the Kruskal–Wallis test (non-parametric ANOVA) then a *post-Hoc* “Scheffe test” was used for pair-wise comparison based on Kruskal–Wallis distribution. Correlation between numerical variables was determined by the Spearman-rho method. Wilcoxon-signed ranks test (non-parametric paired *t*-test) was used to compare two consecutive measures of numerical variables. All tests were two-tailed. A *p*-value < 0.05 was considered significant.

## Results

**Figure 1:**
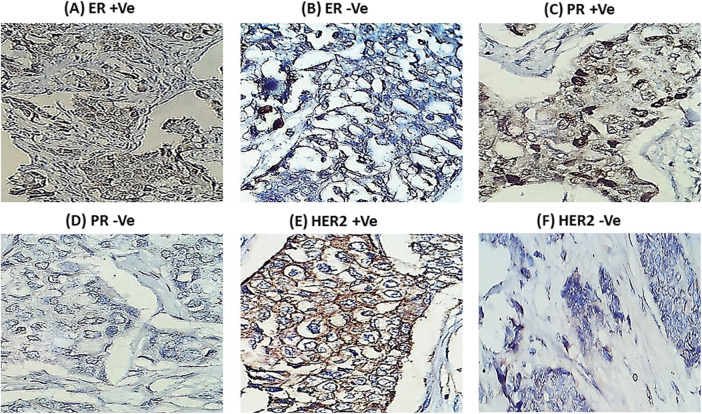
Mmunohistochemistry for hormonal receptors in female breast cancer patients (A) Positive estrogen receptor (ER +Ve). (B) Negative estrogen receptor (ER −Ve). (C) Positive progesterone receptor (PR +Ve). (D) Negative progesterone receptor (PR −Ve). (E) Positive HER-2/neu (HER2 +Ve). (F) Negative HER-2/neu (HER2 −Ve). Black arrows pointed to nuclei and cytoplasm staining sites.

Table 1 shows the characteristics of the 179 BC female patients, and Fig. 1 shows the immunohistochemistry of ER, PR and HER-2/neu.

**Table 1:** The characteristics of 179 breast cancer female patients

Characteristics	Number of patients	% of cases from 179 patients	Pearson chi-square	Likelihood ratio	Significance
**Subtypes**			**134.2**	**142.3**	**0.000**
***Luminal A***	66	37			
***Luminal B***	47	26			
***Her-2/neu***	16	9			
***Basal Like***	50	28			
**Tumor size (T) (cm)**			**80.6**	**83.6**	**0.000**
***T1 > 2 cm***	12	7			
***T2 = 2–5 cm***	136	76			
***T3 > 5 cm***	31	17			
**Lymph node number (N)**			**83.9**	**88.2**	**0.000**
***N0 = 0***	12	7			
***N1 = 1***	135	75			
***N2 = 2***	18	10			
***N3 = 3***	14	8			
**Metastasis (M0)**	179	100			
**TNM stage**			**83.8**	**87.0**	**0.000**
***I***	12	7			
***II***	135	75			
***III***	32	18			
**ER status**			**87.4**	**84.3**	**0.000**
***Negative***	67	37			
***Positive***	112	63			
**PR status**			**71.3**	**29.3**	**0.000**
***Negative***	73	41			
***Positive***	106	59			
**Her-2/neu status**			**26.2**	**30.8**	**0.000**
***Negative***	116	65			
***Positive***	63	35			

**Figure 2:**
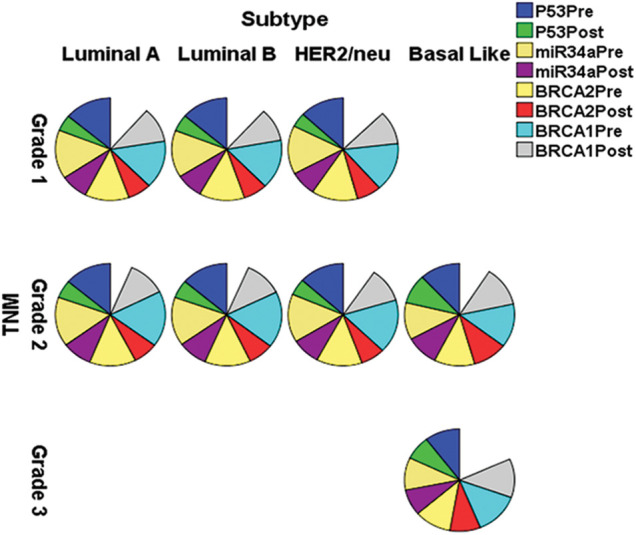
The expression of miR-34a, BRCA1, BRCA2 and p53 in the different subtypes *vs*. clinical stages, pre and post-chemotherapy. T: refers to tumor size, N: node status, M: metastasis.

Table 2 and Fig. 2 reveal that gene fold expression of miR-34a, BRCA1, BRCA2, and p53 is significantly up-regulated (*p <* 0.001) in BC with +ve ER, PR, and HER-2/neu compared to −ve ER, PR, and HER-2 before and after chemotherapy in BC with +ve ER, PR, and HER-2. Before and after chemotherapy, the expression of miR-34a, BRCA1, BRCA2, and p53 is significantly down-regulated (*p <0.001*) in basal-like compared to luminal A and B and HER2/neu subtypes, as well as in stage III compared to stages I and II. Only the expression of BRCA1 was substantially greater in stage I compared to stage II before the treatment of all the biological indicators evaluated.

**Table 2:** The expression of miR-34a, BRCA1, BRCA2 and p53 in Estrogen Receptor (ER), Progesterone Receptor (PR), and Human epidermal growth factor receptor 2 (HER2/neu) female patients in the different subtypes and clinical stages of breast cancer, pre- and post-chemotherapy (Mean ± SD). The numbers between brackets represent the number of patients

	miR-34a		BRCA1		BRCA2		p53	
	Pre	Post	Pre	Post	Pre	Post	Pre	Post
***Estrogen Receptor (ER)***								
**Positive (112)**	−15.03 ± 0.63	−8.47 ± 0.99*	−16.99 ± 1.22	−10.89 ± 1.08*	−13.17 ± 0.57	−7.16 ± 0.79*	−13.46 ± 1.02	−5.50 ± 1.08
**Negative (67)**	−15.87 ± 0.91	−11.82 ± 2.84*	−19.35 ± 1.84	−16.58 ± 3.81	−15.96 ± 1.73	−12.29 ± 3.52*	−15.55 ± 2.12	−10.41 ± 2.28*
***Progesterone Receptor (PR)***								
**Positive (106)**	−15.00 ± 0.64	−8.44 ± 0.98*	−16.97 ± 1.22	−10.90 ± 1.11*	−13.17 ± 0.58	−7.16 ± 0.81*	−13.44 ± 1.03	−5.48 ± 1.07*
**Negative (73)**	−15.81 ± 0.91	−11.58 ± 2.85*	−19.18 ± 1.88	−16.10 ± 3.99	−15.73 ± 1.82	−11.88 ± 3.65*	−15.39 ± 2.10	−10.03 ± 2.31*
***Human epidermal growth factor receptor 2 (HER2/neu)***								
**Positive (63)**	−15.00 ± 0.67	−8.41 ± 1.15*	−17.01 ± 1.25	−10.89 ± 1.28*	−13.24 ± 0.63	−7.25 ± 0.99*	−13.44 ± 1.07	−5.45 ± 1.25*
**Negative (116)**	−15.53 ± 0.88	−10.44 ± 2.73*	−18.34 ± 1.99	−14.17 ± 4.07*	−14.74 ± 1.96	−10.08 ± 3.73*	−14.67 ± 2.00	−8.36 ± 4.08*
***Subtypes***								
**Luminal A (66)**	−15.09 ± 0.61	−8.51 ± 0.79*	−16.91 ± 1.18	−10.86 ± 0.73*	−13.17 ± 0.51	−7.08 ± 0.37*	−13.44 ± 0.97	−5.45 ± 0.77*
**Luminal B (47)**	−14.93 ± 0.67	−8.43 ± 1.22*	−17.10 ± 1.27	−10.92 ± 1.43*	−13.18 ± 0.66	−7.28 ± 1.14*	−13.49 ± 1.08	−5.56 ± 1.39*
**HER2/neu (16)**	−15.22 ± 0.65	−8.34 ± 0.95*	−16.75 ± 1.17	−10.82 ± 0.71*	−13.43 ± 0.50	−7.16 ± 0.22*	−13.29 ± 1.06	−5.11 ± 0.58*
**Basal Like (50)**	−16.11 ± 0.86	−12.99 ± 2.25	−20.23 ± 1.04	−18.55 ± 1.97	−16.81 ± 1.00	−14.04 ± 2.09	−16.31 ± 1.84	−12.21 ± 3.42*
***Clinical Stage***								
**I (12)**	−15.12 ± 0.65	−7.99 ± 0.64*	−15.40 ± 0.40	−10.66 ± 0.65*	−13.25 ± 0.67	−7.03 ± 0.24*	−13.67 ± 1.01	−5.11 ± 0.60*
**II (135)**	−15.20 ± 0.79	−9.16 ± 2.07*	−17.39 ± 1.31	−11.77 ± 2.65*	−13.65 ± 1.36	−8.09 ± 2.61*	−13.83 ± 1.53	−6.48 ± 2.90*
**III (32)**	−16.03 ± 0.84	−12.77 ± 2.23	−20.83 ± 0.52	−19.16 ± 0.81	−16.96 ± 0.62	−14.04 ± 1.88	−16.17 ± 1.99	−11.80 ± 3.71*

Note: * Post-chemotherapy is significant as compared to pre-chemotherapy at the 0.05 level.

**Figure 3:**
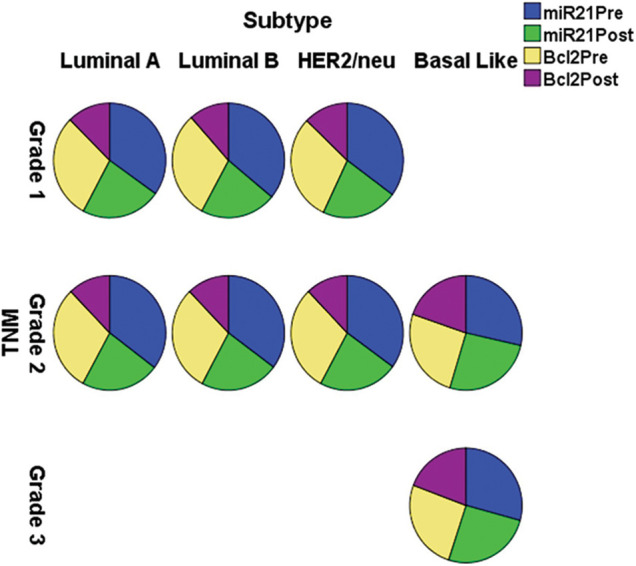
The expression of miR-21 and Bcl-2 in the different subtypes *vs*. clinical stages, pre and post-chemotherapy. T: refers to tumor size, N: node status, M: metastasis

The expression of miR-21 and Bcl-2 is significantly down-regulated (*p <* 0.001) in patients with +ve ER, PR, and HER-2/neu compared to patients with −ve ER, PR, and HER-2 before and after chemotherapy, as shown in Table 3 and Fig. 3. In basal-like compared to luminal A and B and HER2/neu subtypes, as well as in stage III compared to stages I and II, miR-21 and Bcl2 expression is significantly up-regulated (*p <* 0.001).

**Table 3:** The expression of miR-21 and Bcl2 in Estrogen Receptor (ER), Progesterone Receptor (PR), and Human epidermal growth factor receptor 2 (HER2/neu) female patients in the different subtypes and clinical stages of breast cancer, pre- and post-chemotherapy (Mean ± SD). The numbers between brackets represent the number of patients

	miR-21		Bcl2	
	Pre	Post	Pre	Post
***Estrogen Receptor (ER)***				
*Positive (112)*	17.33 ± 1.06	11.06 ± 1.34*	14.73 ± 0.88	5.96 ± 0.51*
*Negative (67)*	19.78 ± 2.23	16.63 ± 4.44	17.41 ± 2.30	11.87 ± 4.76*
***Progesterone Receptor (PR)***				
*Positive (106)*	17.31 ± 1.06	11.06 ± 1.35*	14.73 ± 0.89	5.95 ± 0.51*
*Negative (73)*	19.61 ± 2.22	16.18 ± 4.53	17.18 ± 2.33	11.40 ± 4.84*
***Human epidermal growth factor receptor 2 (HER2/neu)***				
*Positive (63)*	17.36 ± 1.11	11.11 ± 1.47*	14.84 ± 1.00	5.97 ± 0.58*
*Negative (116)*	18.73 ± 2.19	14.25 ± 4.44*	16.21 ± 2.28	9.37 ± 4.67*
***Subtypes***				
*Luminal A (66)*	17.24 ± 1.00	11.03 ± 1.08*	14.60 ± 0.71	5.94 ± 0.40*
*Luminal B (47)*	17.43 ± 1.15	11.11 ± 1.63*	14.88 ± 1.06	5.97 ± 0.63*
*HER2/neu (16)*	17.17 ± 1.01	11.12 ± 0.86*	14.72 ± 0.81	5.98 ± 0.44*
*Basal Like (50)*	20.70 ± 1.72	18.49 ± 3.53	18.34 ± 1.84	13.88 ± 3.78*
***Clinical Stage***				
*I (12)*	17.35 ± 0.92	10.86 ± 0.78*	14.80 ± 0.67	5.88 ± 0.33*
*II (135)*	17.78 ± 1.65	12.17 ± 3.16*	15.26 ± 1.71	7.10 ± 3.14*
*III (32)*	20.57 ± 1.94	18.13 ± 3.87	18.09 ± 1.97	13.56 ± 4.10*

Note: * Post-chemotherapy is significant as compared to pre-chemotherapy at the 0.05 level.

The fold change expression of miR-34a post-chemotherapy is strongly positively correlated with BRCA1, BRCA2, and p53, whereas miR-21 and Bcl2 are negatively correlated (Table 4).

**Table 4:** The correlation (*r*) between the gene fold expression of BRCA1, BRCA2, miR-21, miR-34a, p53 and Bcl2 in breast cancer patients post-chemotherapy *p* < 0.001

		miR34aPost	BRCA1Post	BRCA2Post	p53Post	miR21Post	Bcl2Post
**BRCA1Post**	r	0.862**					
	Sig.	0.000					
**BRCA2Post**	r	0.886**	0.944**				
		Sig.	0.000	0.000				
**p53Post**	Correlation	0.948**	0.852**	0.896**			
	Sig.	0.000	0.000	0.000			
**miR21Post**	r	−0.918**	−0.862**	−0.888**	−0.942**		
		Sig.	0.000	0.000	0.000	0.000		
**Bcl2Post**	r	−0.922**	−0.869**	−0.907**	−0.953**	0.972**	
	Sig.	0.000	0.000	0.000	0.000	0.000	

Note: **Correlation is significant at the 0.01 level. r is a Pearson Correlation.

**Figure 4:**
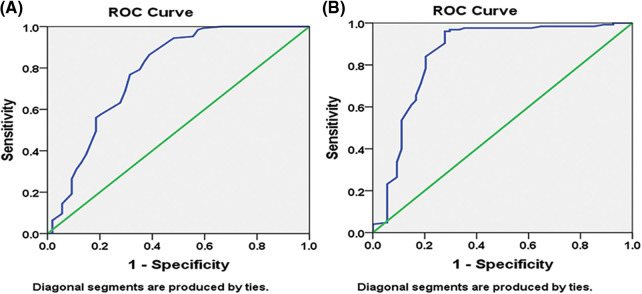
Pre-chemotherapy receiver operating characteristic (ROC) curve: (A) miR-34a (B) miR-21.

With the spearmen correlation coefficient of sensitivity and 1–specificity of (*p* = 0.0001), the diagnostic the threshold analysis to see whether there is a threshold effect in this research indicated no heterogeneity from threshold effect. The 95 percent confidence intervals (CIs) for the cutoff, sensitivity, specificity, and 1–specificity of miR-34a were −15.95, 0.880, 0.593, and 0.407 (95 percent CI: 0.698–0.865), respectively, and the area under the curve (AUC) of Receiver operating characteristic (ROC) was 0.7810.042 (Fig. 4A). The cutoff, sensitivity, specificity, and 1–specificity of miR-21 were 18.44, 0.840, 0.796, and 0.204 (95 percent CI: 0.693–0.865), respectively, with 95 percent CIs of 18.44, 0.840, 0.796, and 0.204 (95 percent CI: 0.693–0.865). The AUC of ROC was 0.8510.038 (Fig. 4B).

## Discussion

Changes in the expression of specific miRNAs have been shown to represent pathological diseases. They are interesting diagnostic indicators for several types of cancer, including BC [26], due to their rematkable stability in human blood [27–29]. miR-21 is one of the most critical miRNAs linked to cell motility and invasiveness in BC cells, resulting in tumor growth and metastasis [30,31], and consequently is considered an oncogene (oncomiR) [32].

The current investigation revealed that at diagnosis, miR-21 was up-regulated in female BC patients, denoting an over-expression [26]. Furthermore, its expression pattern was significantly lower in patients with low-risk hormonal profiles (ER+ve, PR+ve, Her2/neu+ve), compared to patients with ER-ve, PR-ve, Her2-ve profiles. In addition, the miR-21 gene fold expression was highly associated with basal-like subtype and advanced clinical stage III, indicating a poor sensitivity to chemotherapy, which might be ascribed to its role in drug resistance. The multivariate statistical analysis demonstrates that miR-21 may be considered an independent prognostic factor in negative hormonal receptors and advanced BC stage. Patients with a good prognosis presented lower miR-21 expression, and the results showed that it was inversely correlated with BRCA1 and BRCA2 expression while directly correlated with Bcl-2 pre and post-chemotherapy, demonstrating that miR-21 could be considered a significant biological marker to evaluate the response of female BC patients to the applied chemotherapy.

On the other hand, by considering the miR-34a gene expression, the results revealed it was more significant in patients with positive hormonal receptors and minor in the basal-like subtype. Pre- and post-chemotherapy, miR-34a expression was strongly linked with BRCA1 and BRCA2 gene fold expression, as well as p53 gene fold expression. In addition, its expression was down-regulated in stage III *vs*. stages I and II. The p53 network, which is down-regulated in different malignancies, regulates miR-34a transcriptionally [33]. In this line, the level of miR-34a was found to be decreased in different BC cell lines [34,35], verifying that mutations of p53 contribute to lower miR-34a expression.

Reduced cell proliferation, invasion, and increased apoptosis are all linked to ectopic expression of miR-34a in BC cells [36]. In the present study, Bcl-2 expression is inversely correlated with ectopic miR-34a expression, suggesting that miR-34a inhibits BC cell proliferation and migration via downregulating Bcl-2. The increased expression of miR-34a post-chemotherapy shows that this biological marker performs as a suppressor of tumor gene in BC cell growth and migration and owns an antitumor influence, although down-regulated at diagnosis. Moreover, miR-34a correlated directly with the tumor suppressor genes BRCA1, BRCA2 and p53. A miRNA panel has been shown to discriminate malignancies from healthy patients [37] reliably, and serum miR-21 and miR-34a have greater sensitivity in the diagnosis of BC than CEA and CA153 [37,38]. The AUC was utilized as the assessment criterion in the present research to establish the diagnostic performance of miRNAs for BC. The greater the AUC, the better the diagnostic performance [38]. With AUC values of 0.781 ± 0.042 and 0.851 ± 0.038, respectively, our findings demonstrated that miR-34a and miR-21 had considerably higher AUC. miR-34a and miR-21 had 88.0% and 84.0% sensitivity, respectively, and 59.3% and 79.6% specificity, respectively.

## Conclusion

miR-34a and miR-21 may be considered non-invasive molecular biomarkers in diagnosing BC and in evaluating female patients with breast cancer’s reaction to the chemotherapy treatment.

## Data Availability

All the data supporting the conclusions of this manuscript are included within the article. Please contact author for raw data requests.

## Abbreviations

AUC: Area under the curve
BC: Breast cancer
Bcl-2: B-cell lymphoma 2
BRCA: Breast cancer susceptibility gene
DAB: Diaminobenzidine
ER: Estrogen Receptor
HER2: Human epidermal growth factor receptor 2
miR: microRNA
p53: Tumor suppressor protein p53
PR: Progesterone receptor
ROC: Receiver operating characteristic
UTR: Untranslated region
